# Effect of lactate administration on mouse skeletal muscle under calorie restriction

**DOI:** 10.1016/j.crphys.2021.09.001

**Published:** 2021-09-16

**Authors:** Takanaga Shirai, Kazuki Uemichi, Yuki Hidaka, Yu Kitaoka, Tohru Takemasa

**Affiliations:** a Graduate School of Comprehensive Human Sciences, University of Tsukuba, 1-1-1 Tennodai, Tsukuba, 305-8574, Ibaraki, Japan; b Faculty of Health and Sport Sciences, University of Tsukuba, 1-1-1 Tennodai, Tsukuba, 305-8577, Ibaraki, Japan; c Research Fellow of Japan Society for Promotion Science, Japan; d School of Physical Education, Health and Sport Sciences, University of Tsukuba, 1-1-1 Tennodai, Tsukuba, 305-8574, Ibaraki, Japan; e Department of Human Sciences, Kanagawa University, 3-27-1 Rokkakubashi, Kanagawa-ku, Yokohama-shi, Kanagawa, 221-8686 Japan

**Keywords:** Keywards, Lactate, Calorie restriction, mTOR signaling, Autophagy, Skeletal muscle

## Abstract

Calorie restriction (CR) involves a reductions of calorie intake without altering the nutritional balance, and has many beneficial effects, such as improving oxidative metabolism and extending lifespan. However, CR decreases in skeletal muscle mass and fat mass in correlation with the reduction in food intake. Lactate is known to have potential as a signaling molecule rather than a metabolite during exercise. In this study, we examined the effects of the combination of caloric restriction and lactate administration on skeletal muscle adaptation in order to elucidate a novel role of lactate. We first demonstrated that daily lactate administration (equivalent to 1 g/kg of body weight) for 2 weeks suppressed CR-induced muscle atrophy by activating mammalian/mechanistic target of rapamycin (mTOR) signaling, a muscle protein synthesis pathway, and inhibited autophagy-induced muscle degradation. Next, we found that lactate administration under calorie restriction enhanced mitochondrial enzyme activity (citrate synthase and succinate dehydrogenase) and the expression of oxidative phosphorylation (OXPHOS) protein expression. Our results suggest that lactate administration under caloric restriction not only suppresses muscle atrophy but also improves mitochondrial function.

## Highlights

• CR improves oxidative metabolism but decreases muscle mass.
• Lactate administration under caloric restriction inhibits muscle mass loss.
• Lactate administration not only improves oxidative metabolism, but also activates mTOR signals and prevents atrophy under CR.

## Graphical abstract

**Figure:**
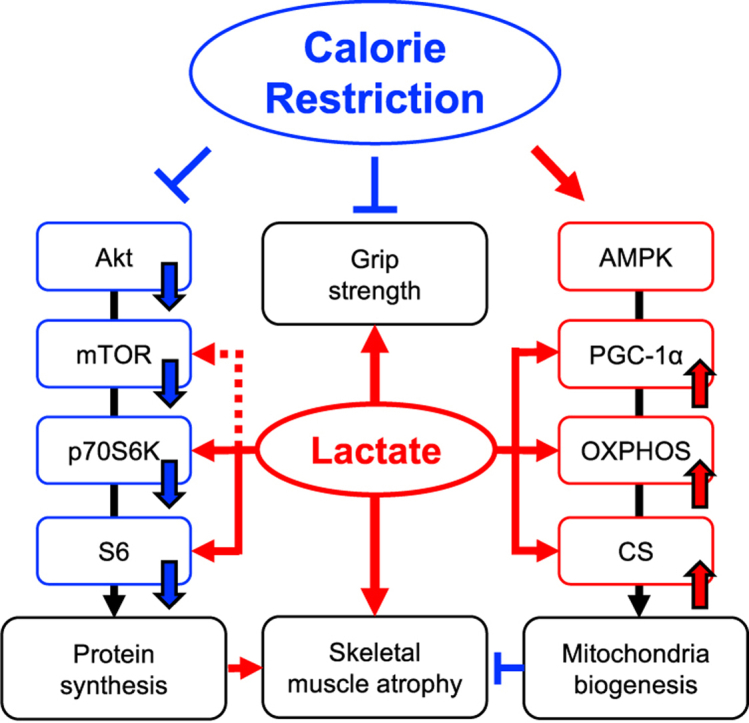

## 1 Introduction

Skeletal muscle accounts for 30%–40% of the human body (Janssen et al., 2000) and is a very unique tissue with high plasticity. While skeletal muscle mass increases with resistance exercise and proper nutrition (Damas et al., 2016; Dickinson et al., 2011), it decreases with inactivity (Gao et al., 2018). Skeletal muscle mass is regulated by the balance of protein synthesis and breakdown. Mammalian/mechanistic target of rapamycin (mTOR) signaling plays an important role in the maintenance of skeletal muscle mass (Bodine et al., 2001; Uemichi et al., 2021). It has been demonstrated that this signaling pathway is activated by overload stimuli such as resistance exercise and the intake of amino acids (Damas et al., 2016; Dickinson et al., 2011; Shirai et al., 2021).

Calorie restriction (CR) is a method of reducing total caloric intake by intermittently restricting food intake. CR has been associated with improved health, increased lifespan, and decreased morbidity and mortality in animal studies (Buschemeyer et al., 2010; Ikeno et al., 2006). Calorie control also has many benefits in humans, including cardiovascular status, weight loss, insulin sensitivity, diabetes control, cognitive function, and cancer prevention(Colman et al., 2009; Mattison et al., 2012). Whereas, CR is not only decreases the incidence of aging-related diseases such as cardiovascular diseases and diabetes, but also reduce body weight, muscle and fat mass (Sipe et al., 2017). Under CR, mTOR signaling is inactivated and autophagy, the protein degradation mechanism, is activated (Nonaka et al., 2017). While CR also results in a reduction in skeletal muscle mass, it has beneficial effects on muscle quality, such as increasing the amount of mitochondria in skeletal muscle (Kitaoka et al., 2016a). As for athletes, CR is a conditioning method and has been adopted by many weight-class sports such as judo, boxing, and weightlifting, and aesthetic athletes such as rhythmic gymnasts and figure skaters. However, CR reduces not only fat mass but also skeletal muscle mass. Therefore, there is a requirement for a weight loss method that can maintain sports performance without reducing skeletal muscle mass as much as possible.

In few decades, lactate, the end product of the glycolytic system, has been considered a waste product that causes fatigue, but we have come to recognize lactate as an important metabolic substrate (Brooks, 2018). Recently, it has been reported that lactate, which is produced by glycolysis during aerobic exercise, acts as a signaling molecule (Allen and Westerblad, 2004; Rawat et al., 2019). Previous studies have reported that lactate administration increases the expression of genes involved in oxidative metabolism in skeletal muscle, especially mitochondria-related genes (Kitaoka et al., 2016b), and that the regulation of lactate production by drug administration alters skeletal muscle adaptation (Hoshino et al., 2015; Percival et al., 2015). These studies report that elevation of blood lactate by sodium bicarbonate enhances the expression of mitochondrial genes, while suppression of blood lactate levels by dichloroacetic acid decreases the expression of mitochondrial genes. Lactate administration has also been reported to enhance not only signaling in oxidative metabolism (Takahashi et al., 2019), but also protein synthesis in skeletal muscle (Cerda-Kohler et al., 2018). Lactate administration increases both the phosphorylation of p70S6 kinase (p70S6K), which is downstream of mTOR signaling, and the expression of extracellular signal-regulated kinase (ERK1/2) (Cerda-Kohler et al., 2018). These findings suggest that lactate acts as a hypertrophic triggering molecule in skeletal muscle.

Based on the above background, lactate may not only increase mitochondrial biogenesis, but also act as a trigger for anabolic responses, suppressing catabolic responses and skeletal muscle mass loss under CR. Although heat stress and resistance exercise are generally considered to causes similar results, we followed previous studies and focused on examining anabolic and mitochondrial responses under CR in order to verify the mechanism of direct lactate signaling. We hypothesized that lactate suppresses the decrease in protein synthesis and rescues the decrease in muscle mass under CR.

## 2 Material and methods

### 2.1 Animals

All experimental procedures performed in this study were approved by the Institutional Animal Experiment Committee of the University of Tsukuba (20–407). Male ICR mice aged 7–8 weeks (Tokyo Laboratory Animals Science Co., Tokyo, Japan) were used in this study. The mice were kept in temperature (22 ± 2 °C) and humidity (55 ± 5%)-controlled facilities under a 12/12 h light/dark cycle with *ad libitum* access to food (protein:fat:carbohydrate = 23.1%:5.1%:71.8%: Oriental, Japan) and water. The mice were divided into three groups; phosphate-buffered saline (PBS)-administered group (Control; n = 7), PBS-administered CR group (CR; n = 7), and lactate-administered CR group (CR + Lac; n = 8). The animals were intraperitoneally administered PBS or 1 g/kg of body weight of sodium lactate once a day at 19:00 h for 14 consecutive days. After 1 week of acclimation, mice in the CR group were given 60% of the average amount of food eaten by each mouse for 1 week acclimation. Mice in the CR group were fed 60% of the diet daily at 19:00 h, and food intake in both groups was measured every 24 h from the start of the experiment. Fourteen days after the experiments, the mice were anesthetized and the muscles were excised, weighed, quickly frozen in liquid nitrogen, and stored at −80 °C until for further analyses.

### 2.2 Grip strength test

Forelimb grip strength was measured using a grip strength meter (GPM-100; Melquest, Japan), as previously described (Fujiwara et al., 2019; Yoshizawa et al., 2017). The forelimbs of the mice were allowed to grab a horizonal bar mounted on the gage and the tail was slowly pulled back by an experimenter, Tension (in kilograms) was recorded when the mouse released its grip. Measurements were repeated in five trials, with the mean value being used as the mean grip strength for data analysis.

### 2.3 Cross-sectional area (CSA) quantification

The gastrocnemius muscle was covered with optimal cutting temperature compound (Sakura Finetek, Tokyo, Japan), and then quickly frozen in liquid nitrogen-cooled isopentane and stored at −20 °C until sectioning. The frozen muscle was sectioned at a thickness of 10 μm, air-dried, and stored at −20 °C. Images were captured with an Olympus DP-74 microscope (Tokyo, Japan) and CSA analysis was carried out using the Image J (NIH) software.

### 2.4 Western blotting

Excised gastrocnemius muscles were immediately frozen in liquid nitrogen and total muscle protein was extracted by lysis buffer containing 50 mM HEPES (pH: 7.6), 150 mM NaCl, 10 mM EDTA, 10 mM Na_4_P_2_O_7_, 10 mM NaF, 2 mM Na_3_VO_4_, 1% (vol/vol) NP-40, 1% (vol/vol) Na-deoxycholate, 0.2% (wt/vol) SDS, and 1% (vol/vol) complete protease inhibitor cocktail. Protein concentrations were measured using a Protein Assay Bicinchoninate Kit (Nacalai Tesque Inc., Kyoto, Japan). Before SDS-PAGE, an aliquot of the extracted protein solution was mixed with an equal volume of sample loading buffer containing 1% (vol/vol) 2-mercaptoethanol, 4% (wt/vol) SDS, 125 mM Tris-HCl (pH: 6.8), 10% (wt/vol) sucrose, and 0.01% (wt/vol) bromophenol blue. The mixture was then heated at 97 °C for 3 min. Ten micrograms of each protein sample was separated on an SDS-polyacrylamide gel and electrically transferred to an ImmunoBlot PVDF membrane (Bio-Rad Laboratories, Hercules, CA, USA). The blot was blocked by Blocking One (Nacalai Tesque Inc.,) for 1 h at room temperature and incubated with primary antibodies overnight at 4 °C in TBS containing 0.1% Tween-20. After overnight incubation, membranes were incubated with horseradish peroxidase-conjugated secondary antibody for 60 min at room temperature. Signals were detected using Immunostar Zeta or LD (Wako Chemicals, Osaka, Japan), quantified with C-Digit (LI-COR Biosciences, Lincoln, NE, USA), and expressed in arbitrary units.

### 2.5 Primary antibodies for western blotting

The following primary antibodies were used for western blotting: protein kinase B (Akt) (#9272), p-Akt (#4060S), p70 S6 kinase (p70S6K) (#9202), p-p70S6K (#9205), S6 ribosomal protein S6 (S6) (#2217), p-S6 (#4858S), 4E-binding protein 1 (4E-BP1) (#9452), p-4E-BP1 (#9459), and MAP1LC3 (Microtubule-associated protein 1 light chain 3 (LC3) (4108; all from Cell Signaling Technology, Danvers, MA, USA), Muscle Atrophy F-box (MAFbx) (sc-33782; Santa Cruz), Muscle RING-Finger Protein (MuRF1) (sc-32920; Santa Cruz), p62 (SQSTM1) (PM045, MBL), ubiquitin (sc-166553; Santa Cruz) AMP-activated protein kinase (AMPK) (#2532; Cell Signaling Technology), p-AMPK (#2531; Cell Signaling Technology), PGC-1α (516557; Millipore) and oxidative phosphorylation (OXPHOS) (ab110413; Abcam, Cambridge, UK).

### 2.6 Enzyme activity

Maximal activity of citrate synthase (CS) in whole plantaris muscle was determined using the standard procedure as described previously (Takeda et al., 2018).

### 2.7 Succinate dehydrogenase (SDH) staining

SDH staining was performed as previously described (Lee et al., 2018). Gastrocnemius muscle was used in SDH staining. Tissue slides were incubated in a solution containing 0.2 M phosphate buffer (pH 7.6), 100 mM sodium succinate, and 1.2 mM nitro blue tetrazolium for 90 min at 37 °C. After removing unbound nitro blue tetrazolium by washing with PBS solution, slides were placed inside a chemical hood until dry, and then a cover glass was applied with a drop of Permount histological mounting medium (SereCare Life Science Inc, Milford, MA, USA). SDH density was measured by Image J software (NIH).

### 2.8 Statistical analysis

Data are shown as mean ± standard error (SE). Student's *t*-test was used for comparisons between the two groups. One-way analysis of variance (ANOVA) followed by Tukey's post hoc test was conducted for all measurements. The GraphPad Prism7 software (GraphPad, Inc., San Diego, CA, USA) was used for all statistical calculations, and the significance level was set to p < 0.05 for all cases.

## 3 Results

### 3.1 Animal characteristics

**Fig. 1:**
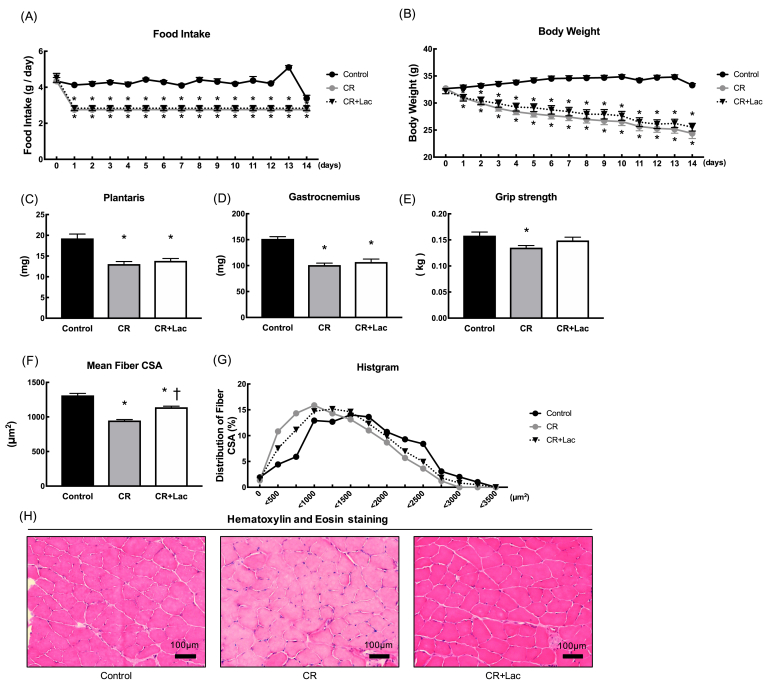
Animal characteristics. (A) Food intake, (B) body weight, (C) plantaris weight, (D) gastrocnemius weight, (E) grip strength, (F) mean fiber CSA, (G) histogram of fiber CSA and (H) Hematoxylin and Eosin staining. Values represent mean ± SE (n = 7–8 per group). *P < 0.05 vs. Control, ^†^P < 0.05 vs. CR by one-way ANOVA.

Animal characteristics are shown in Fig. 1. Food intake was significantly lower in the CR and CR + Lac groups (Control: 4.25 ± 0.1 g, CR: 2.75 ± 0.1 g, CR + Lac: 2.83 ± 0.1 g) (Fig. 1A). Body weight was significantly lower in the CR and Lac + CR groups from day 1 of the experiment (Fig. 1B). Wet weight of plantaris and gastrocnemius muscles were significantly lower in the CR and CR + Lac groups than in the Control group at the end of the experiment (Fig. 1C and D). Grip strength was significantly lower in the CR group than in the Control group (Fig. 1E). The CSA of muscle fibers was significantly lower in the CR and Lac + CR groups than in the Control group. The transverse area of myofibers was significantly higher in the Lac + CR group than that in the CR group (Fig. 1F). The histogram of the CSA of myofibers was shifted to the left in the CR group compared with that in the control group, while the histogram of the Lac + CR group was shifted to the right compared with that in the CR group (Fig. 1G). Myofiber images of each group are shown in Fig. 1H.

### 3.2 Anabolic proteins

**Fig. 2:**
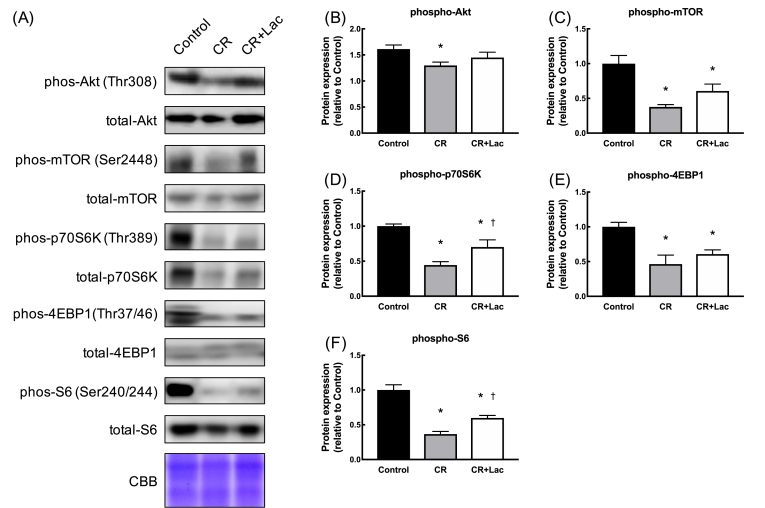
Effects of daily lactate administration under CR on anabolic proteins in skeletal muscle. (A) Band images, (B) phospho Akt, (C) phospho mTOR, (D) phospho p70S6K, (E) phospho 4EBP1, and (F) phospho S6. Values represent mean ± SE (n = 7–8 per group). *P < 0.05 vs. Control, ^†^P < 0.05 vs. CR by one-way ANOVA.

The phosphorylation levels of molecular signals of the Akt/mTOR cascade related to muscle protein synthesis were quantified. The phosphorylation levels Akt, mTOR, p70S6K, 4EBP1, and S6 were significantly lower in the CR and Lac + CR groups than those in the Control group. In contrast, the phosphorylation levels of p70S6K and S6 were significantly higher in the Lac + CR group than those in the CR group. These results indicated that lactate administration under CR increased the phosphorylation levels of p70S6K and S6 (Fig. 2A, B, C, D, E, F).

### 3.3 Catabolic proteins

**Fig. 3:**
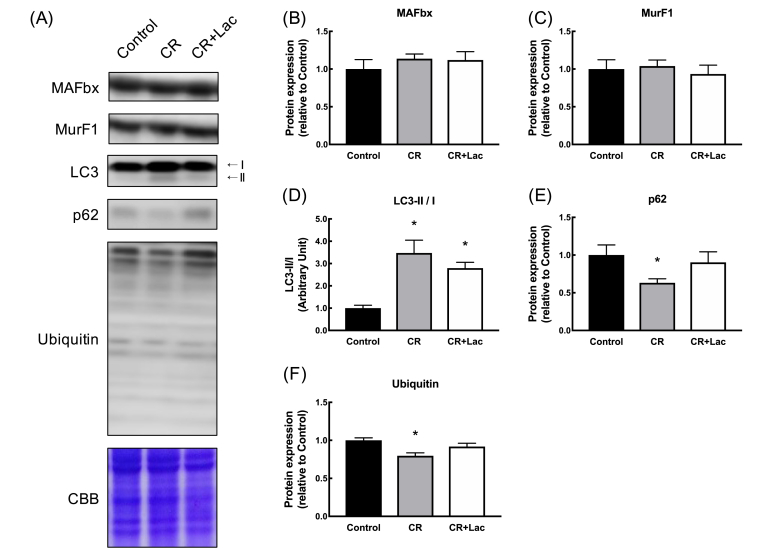
Effects of daily lactate administration under CR on catabolic proteins in skeletal muscle. (A) Band images, (B) MAFbx, (C) MuRF1, (D) LC3-Ⅱ/Ⅰ, (E) p62 and (F) ubiquitinated proteins. Values represent mean ± SE (n = 7–8 per group). *P < 0.05 vs. Control by one-way ANOVA.

The expression levels of proteins related to the ubiquitin–proteasome system and autophagy–lysosome system, which are typical signals related to muscle protein degradation, were measured. The expression levels of MAFbx and MuRF1 were not significantly different between the groups (Fig. 3B and C), whereas increased autophagy flux (i.e., a combination of increased LC3-II/LC3-I ratio and decreased p62 protein content) was observed in the CR group (Fig. 3D and E). The expression levels of ubiquitinated proteins were significantly lower in the CR group than those in the control group (Fig. 3F).

### 3.4 Mitochondria–related proteins and enzyme activity

**Fig. 4:**
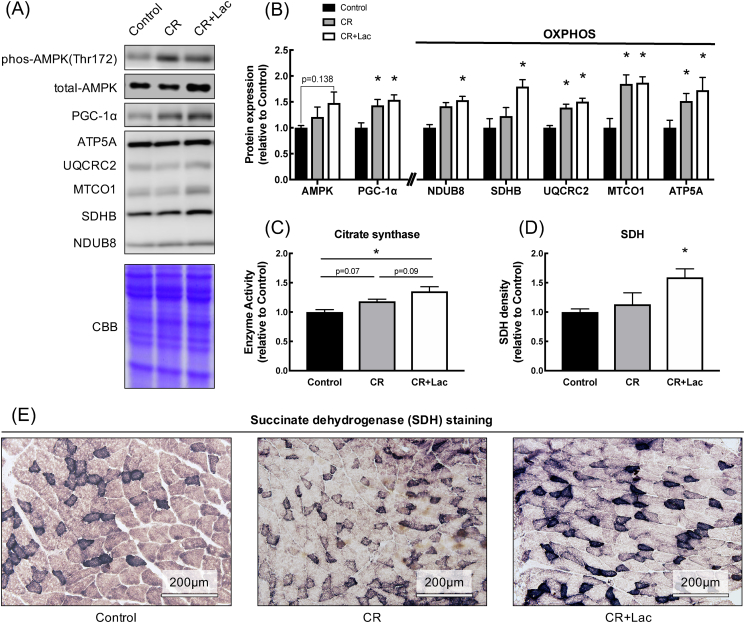
Effect of daily lactate administration under CR on mitochondrial protein expression and enzyme activity in skeletal muscle. (A) metabolism and mitochondria related protein band images, (B) metabolism and mitochondria related proteins, (C) citrate synthase activity, (D) SDH density and (E) SDH staining image. Values represent mean ± SE (n = 7–8 per group). *P < 0.05 vs. Control by one-way ANOVA.

To confirm the effect of CR on mitochondria, we measured protein expression levels of AMPK, oxidative phosphorylation (OXPHOS), CS activity, and enzyme activity by SDH staining. The protein expression levels of AMPK was not different between the group. PGC-1α, which is master regulator of mitochondria and UQCRC2, MTCO1 and ATP5A which is mitochondrial chain complex were significantly higher in the CR and Lac + CR groups than that in the control group. NDUB and SDHB were significantly higher in the Lac + CR group than that in the control group (Fig. 4 A and B), and CS activity was significantly higher in the Lac + CR group that in the control group (Fig. 4C). The enzyme activity by SDH staining was significantly higher in the Lac + CR group than that in the CR group. SDH staining images are shown in Fig. 4E (Fig. 4D and E).

## 4 Discussion

In the present study, we investigated the effects of lactate administration on skeletal muscle adaptation and its molecular mechanisms under CR, focusing on muscle anabolic and catabolic signaling, and mitochondria. Our data suggested that lactate administration suppressed the decrease in myofiber CSA and enhanced mitochondrial function under CR.

In this study, the amount of food was restricted to 40% of the average food intake of each individual mouse during the acclimation period. As a result, body weight decreased significantly from the beginning to the end of the experimental period and the reduction in muscle weight due to CR was not rescued by lactate administration. However, lactate administration prevented the decreases in grip strength and CSA due to CR. These results indicate that lactate administration suppressed the decrease in muscle strength caused by CR.

The expression levels of p70S6K and S6, proteins related to muscle protein synthesis, were also decreased by CR but were rescued by lactate administration. Previous studies reported that lactate administration enhances muscle protein synthesis signals in mouse skeletal muscle (Cerda-Kohler et al., 2018) and that lactate administration to C2C12 enhances protein synthesis signals in a concentration-dependent manner (Oishi et al., 2015). Our data showed that lactate administration increased the phosphorylation levels of p70S6K and S6 in calorie–restricted mice, while caloric restriction decreased the synthetic signal. Interestingly, lactate administration did not change the phosphorylation levels of mTOR, but had a direct effect on p70S6K and S6, which are usually acknowledged to be downstream effectors of mTOR. mTOR is a common molecule in two functionally distinct multiprotein signaling complexes called mTORC1 and mTORC2 (Saxton and Sabatini, 2017). mTORC1 consists of mTOR, Raptor, mLST8 and PRAS40, whereas the core components of mTORC2 include mTOR, Rictor, mLST8 and mSIN1. Generally, mTORC1 plays a role in protein synthesis regulation in cells, while there is minimal information available about the role of mTORC2 in protein synthesis regulation. Protein synthesis is facilitated by mTORC1 via phosphorylation of its direct substrates p70S6K and 4E-BP1. Particularly, Raptor, a component of mTORC1, is known to be an important protein for muscle hypertrophy. Previous studies have shown that Raptor, but not mTOR, is essential for muscle hypertrophy induced by mechanical overload. In addition, Rictor, a protein that is a component of mTORC2, has also been shown to be involved in muscle hypertrophy (Ogasawara et al., 2020; You et al., 2019). Therefore, a more detailed study that takes into account for the each member of mTOR complex is needed to investigate the potential effects of lactate on mTOR signaling. These results suggest that lactate administration may have stimulated those proteins by a pathway other than mTOR, such as through mitogen activated protein kinase (MAPK). p70S6K expression is known to be strongly correlated with CSA (Baar and Esser, 1999), and our results are similar to those in previous studies that also showed that a decrease in grip strength was inhibited by lactate administration (Baar and Esser, 1999). Autophagy flux, which is related to the degradation of muscle protein (Mammucari et al., 2007), was significantly higher in the CR group than in the control group. Our results suggest that muscle protein degradation was enhanced only in the CR group, not in the CR + Lac group. Furthermore, the expression levels of MAFbx and MuRF1, which are important factors in the ubiquitin–proteasome system related to degradation of muscle protein (Zhao et al., 2007), did not differ between the groups. In contrast, levels of ubiquitin-labeled proteins were significantly lower in the CR group than in the control and CR + Lac groups. In the present study, the degradation of muscle proteins by CR involved degradation by the autophagy–lysosome system, not by the ubiquitin–proteasome system (Chen et al., 2019), and lactate administration had no effect on such degradation. These results indicate that lactate administration suppresses the decreased in muscle protein synthesis signaling and degradation by the autophagy-lysosome system under CR. Our data on synthesis- and degradation-related proteins suggest that CR decreases CSA by decreasing muscle protein synthesis and increasing autophagy-induced degradation of muscle protein. On the other hand, the data suggest that the administration of lactate inhibits the decrease in CSA through activation of the muscle protein synthesis factors p70S6K and S6.

In human and rat studies, they were reported that CR enhances mitochondrial biogenesis and increases mitochondrial transcription factors and mtDNA (Civitarese et al., 2007; Kitaoka et al., 2016a); therefore, CR is thought to induce the adaptation of skeletal muscle to efficiently generate energy under conditions of low caloric intake. In addition, lactate administration or oral intake of lactate have been reported to improve mitochondrial enzyme activity and enhance mitochondrial function (Takahashi et al., 2019, 2020). In the present study, we found that lactate administration under CR increased the activity of mitochondrial respiratory chain complex proteins and mitochondrial enzymes, CS and SDH. Lactate administration has also been shown to increase the activity of mitochondrial enzymes in plantaris and soleus muscles (Takahashi et al., 2019). Additionally, CR enhances mitochondrial biogenesis and increases the number of mitochondria in skeletal muscle. CR is thought to enhance mitochondrial biogenesis and increase the number of skeletal muscle mitochondria; 30–40% CR in WT mice increased the expression of PGC-1α and mtDNA in the heart (Nisoli et al., 2005). In a 3-month CR study in humans, TFAM and mtDNA expression was also increased in skeletal muscle (Civitarese et al., 2007). The mitochondrial biogenesis induced by these CRs may be an adaptation of skeletal muscle to efficiently generate energy under low food intake conditions. The mitochondrial biogenesis induced by these CRs is thought to be an adaptation of skeletal muscle to efficiently generate energy under conditions of low food intake. Our results were obtained by combining two methods for enhancing mitochondrial adaptation, CR and lactate administration, revealing that lactate administration enhanced mitochondrial biogenesis even under CR.

CR is widely known to have beneficial effects on health, but it also has the negative effect of reducing skeletal muscle mass. Daily administration of lactate prevented the CR-induced decrease in muscle strength and enhanced mitochondrial adaptation. These results strongly suggest that lactate is not only an important factor in mitochondrial adaptation, but also an important signaling molecule that activates muscle hypertrophy signaling. In future study, it will be necessary to examine the combination of lactate with resistance exercise and amino acid intake to increase muscle size, and to study the role of lactate on muscle size and the signaling molecules that regulate it. Additionally, the role of lactate in enhancing mitochondrial function may be clarified by considering the required amount of lactate and its prescription, as well as the amount required to induce physical adaptation to exercise.

## Funding

This work was supported by 10.13039/501100001691JSPS KAKENHI grants 20J15222.

## CRediT authorship contribution statement

**Takanaga Shirai:** Conceptualization, Investigation, Validation, Data curation, Writing – original draft, contributed to the artwork, drafted the paper, all authors approved the final version of the manuscript. **Kazuki Uemichi:** Investigation, Data curation, all authors approved the final version of the manuscript. **Yuki Hidaka:** Investigation, all authors approved the final version of the manuscript. **Yu Kitaoka:** Data curation, Validation, Writing – review & editing, all authors approved the final version of the manuscript. **Tohru Takemasa:** Conceptualization, Supervision, Writing – review & editing, Conceptualization, Supervision, Writing – review & editing, Writing – original draft, contributed to the artwork, drafted the paper, all authors approved the final version of the manuscript.

## Declaration of competing interest

The authors declare that they have no known competing financial interests or personal relationships that could have appeared to influence the work reported in this paper.
